# Identification of α-glucosidase inhibitory phytochemicals from *Erythrina crista-galli* using combined experimental and computational methods

**DOI:** 10.1186/s13065-026-01785-2

**Published:** 2026-04-10

**Authors:** Herlandita Rona Anggraeni, Abd. Wahid Rizaldi Akili, Muhammad Habibul Ikhsan, Ari Hardianto, Jalifah Latip, Tati Herlina

**Affiliations:** 1 Department of Chemistry, Faculty of Mathematics and Natural Science, Padjadjaran University, Jatinangor, Sumedang, 45363 West Java Indonesia; 2 Department of Chemical Sciences, Faculty of Science and Technology, Universiti Kebangsaan Malaysia (UKM), 46300 Bangi, Selangor Malaysia

**Keywords:** *Erythrina crista-galli*, Alpha-glucosidase, Antidiabetic, Molecular docking, Molecular dynamics

## Abstract

α-Glucosidase inhibition is an important therapeutic strategy for managing type 2 diabetes. This study aims to identify compounds responsible for the α-glucosidase inhibitory activity of *Erythrina crista-galli* stem bark extract. The ethanol extract shows strong antioxidant activity and the highest α-glucosidase inhibitory activity among the samples tested. LC-MS/MS analysis tentatively identified 36 flavonoid constituents, which were subsequently evaluated through molecular docking against the crystal structure of isomaltase (PDB ID: 3A4A). When compared with isomaltose, the natural substrate of the enzyme, two compounds exhibited more favorable predicted binding affinities and engaged key catalytic residues within the active site, suggesting their potential to act as competitive inhibitors. Molecular dynamics simulations over 500 ns showed that isovitexin-2ʹʹ-β-ᴅ-glucopyranoside (**17**) has the lowest MMGBSA binding energy and stable interactions with catalytic residues. ADMET analysis indicated good solubility, intestinal absorption, limited permeability, and low toxicity for isovitexin-2ʹʹ-β-ᴅ-glucopyranoside (**17**). This work provides a scientific foundation for further exploration of *E. crista-galli* and its constituent flavonoids in the development of nutraceutical or pharmaceutical strategies for diabetes management.

## Introduction

Diabetes mellitus is a metabolic disease that leads to high blood glucose caused by the metabolism of carbohydrates, fats, and proteins [1]. As reported by the Diabetes Atlas 2025, 589 million people are living with diabetes worldwide, and the number is estimated to reach 853 million by 2050. Diabetes related complications are also linked with over 3.4 million deaths [2]. Classification of diabetes is broadly divided into two types: type 1 diabetes (T1D), an autoimmune disease where the body is unable to make insulin, and type 2 diabetes (T2D), marked by insulin resistance and a relative insulin deficiency [3]. Among them, type 2 diabetes is the most common, covering over 90% of the global diabetes population [2]. Treatment of hyperglycaemia is usually oral hypoglycemic drugs with lifestyle measures including exercise and dietary control [4, 5]. At present, there are multiple kinds of drugs for diabetes treatment, such as insulin, biguanides, sulfonylureas, thiazolidinediones, α-glucosidase inhibitors (AGIs), glucagon-like peptide-1 (GLP-1) receptor agonists, and sodium-glucose co-transporter 2 (SGLT2) inhibitors [6].

 α-Glucosidase inhibitors (AGIs) delay the digestion of carbohydrates in the small intestine and alleviate the rise of blood glucose after meals. The clinically applied AGIs are acarbose, miglitol, voglibose, and 1-deoxynojirimycin (DNJ) [7]. These drugs are well tolerated overall, but the local gastrointestinal adverse effects may lead to patient noncompliance with the therapy and drug resistance [8, 9]. For this reason, an intensive search for safer and more potent α-glucosidase inhibitors (AGIs) with fewer side effects is going on and natural products, especially bioactive compounds of medicinal plants and foods, have received considerable attention. According to previous reports, flavonoids, tannins, and triterpenes are potential candidates for future AGIs [10].

*Erythrina crista-galli* L. (Fabaceae) is a seasonal flowering species belonging to the genus *Erythrina*, which comprises approximately 130 species distributed in tropical and subtropical regions. Native to South America, this species is recognized for its rich phytochemical composition, particularly erythrinian alkaloids, flavonoids, and other phenolic compounds found in various parts of the plant [11]. Traditionally, *E. crista-galli* has been used in ethnomedicine for the treatment of rheumatism, hepatitis, and inflammatory disorders. Previous pharmacological studies have reported that extracts and isolated compounds from this species exhibit anticancer, phytoestrogenic, antimalarial, antioxidant, and anti-inflammatory activities. These biological effects are largely associated with its diverse secondary metabolites, highlighting its potential therapeutic value [12].

Several *Erythrina* species have been reported to contain prenylated flavonoids with promising biological activities. Previous studies indicate that compounds such as Eryvarin M, Eryvarin H, and Neobavaisoflavone exhibit α-glucosidase inhibitory potential and show favorable binding interactions in molecular docking evaluations. These findings highlight the relevance of *Erythrina* flavonoids as potential AGIs. However, despite the phytochemical richness of the genus, the chemical composition and α-glucosidase inhibitory mechanism of *E. crista-galli* have not been systematically explored [13]. Considering the strong enzyme-inhibitory potential reported in related *Erythrina* species, a comprehensive investigation integrating metabolite profiling with molecular-level interaction analysis remains lacking.

Therefore, this study aims to evaluate the α-glucosidase inhibitory activity of *E. crista-galli* stem bark extract and to identify the key metabolites contributing to the observed activity through an integrated strategy involving LC-MS/MS profiling, molecular docking, molecular dynamics simulation, and ADMET prediction. This approach enables mechanistic elucidation of ligand-enzyme interactions and provides molecular insight into the bioactive constituents underlying the extracts antidiabetic potential.

## Materials and methods

### Plant material

The stem bark of *E. crista-galli* L. was collected in August 2024 from Jalan Sersan Bajuri, West Java, Indonesia. The collection was conducted during the dry season. Taxonomic identification of the plant material was performed by Joko Kusmoro at the Department of Agronomy, Faculty of Agriculture, Padjadjaran University. A voucher specimen (No. 1020) was deposited for future reference. The collection and utilization of plant material were carried out in accordance with relevant national and international guidelines and regulations.

### Extraction and fractionation

*E. crista-galli* stem bark powder (2.5 kg) was extracted using the maceration method with ethanol for 3 × 24 h. The ethanol extract was then filtered and evaporated to produce a concentrated ethanol extract. This concentrated extract was then partitioned using *n*-hexane, ethyl acetate, and *n-*butanol, resulting in *n*-hexane, ethyl acetate, and *n-*butanol fractions.

### Inhibition α -glucosidase assay

A tube containing 5 µl of sample dissolved in DMSO at varying concentrations was added to p-NPG (250 $\:{\upmu\:}$l, 3 mM) and 495 µl of 100 mM phosphate buffer solution (pH 7.0). The reaction mixture was incubated for 5 min at 37 °C, then 250 µl of α-glucosidase (0.065 U/ml) was added and incubated again for 15 min. Add 1 ml of 0.2 M Na_2_CO_3_ solution to stop the reaction. The amount of p-nitrophenol released is measured at λ 400 nm to measure the inhibitory effect on α-glucosidase activity. To correct for background absorbance, the enzyme is replaced with 250 µl of phosphate buffer. The final concentration of DMSO in the reaction mixture was 0.5% (v/v). A negative control containing the same volume of DMSO without a sample was included to account for any potential solvent-related effects. The percentage inhibition of α-glucosidase and antioxidant activity was calculated using the following formula: The percentage of inhibition was calculated using the formula:

$$\% {\text{ }}Inhibition = \left\lfloor {\frac{{(A - B)}}{A}} \right\rfloor \; \times \,100$$

where A is the absorbance of the control and B is the absorbance with the sample. *IC*_50_ values were obtained from inhibition data using logarithmic regression. Quercetin was used as the positive control [14].

### Antioxidant activity

The method for testing antioxidant activity using the 2,2-diphenyl-1-picrylhydrazyl (DPPH) assay involves the following steps: A 0.0004 M DPPH solution was prepared by dissolving 4.0 mg of DPPH in 25 mL of methanol in a volumetric flask. The solution was stored in the dark and covered with aluminium foil. The extracts (1 mg) were dissolved in methanol and diluted to 1 mL to obtain a 1000 ppm stock solution. This stock was further diluted to prepare a series of concentrations. Each concentration was transferred into a dark vial and mixed with 60 µL of 0.0004 M DPPH solution. The mixtures were homogenized and incubated for 30 min in the dark. Absorbance was measured at 515 nm [15].

### Metabolite profiling

Phytochemical analysis of compounds in the stem bark of *E. crista-galli* was performed using LC-MS/MS (Liquid Chromatography–Tandem Mass Spectrometry). The ethanol extract obtained from partitioning was prepared for analysis. The LC-MS/MS system consisted of an Agilent 1200 series (equipped with a binary pump, autosampler, and column oven) coupled to a 6520 Quadrupole Time-of-Flight (QTOF) mass spectrometer, capable of operating in both positive and negative ionization modes.

Compound separation was conducted at a column temperature of 40 °C using a mobile phase composed of water with 0.1% formic acid and acetonitrile. Each sample was analyzed for 30 min, with a 2-min equilibration period before sequential injections. The injection volume was 2 µL, and the flow rate was maintained at 0.25 mL/min. Before analysis, samples were filtered through a 0.22 μm pore size syringe filter. The mass spectrometer operated under Electrospray Ionization (ESI) mode, with a gas temperature of 325 °C, gas flow rate of 11 L/min, and nebulizer pressure of 35 psi [16].

LC–MS/MS raw data were processed using MassLynx software (version 4.1, Waters, USA). Putative compound identification was achieved by matching the molecular formulas predicted by the software with an in-house database consisting of 729 secondary metabolites previously described in species of the genus. The identification was considered tentative and based on accurate mass measurement and literature comparison. This study was qualitative in nature and no absolute or relative quantification was performed.

### Molecular docking

The molecular docking study was conducted using the crystal structure of isomaltase from *Saccharomyces cerevisiae* (PDB ID: 3A4A), obtained from the Protein Data Bank. Protein preparation was performed using BIOVIA Discovery Studio. The native ligand was separated from the receptor, and the protein structure was refined by removing crystallographic water molecules and irrelevant heteroatoms to ensure proper docking conditions. To validate the docking protocol, a redocking procedure was carried out using the co-crystallized ligand. The reliability of the docking method was assessed by calculating the root-mean-square deviation (RMSD) between the experimentally observed and redocked ligand conformations. The protocol was considered valid when the RMSD value of the best-ranked pose was $\:\le\:$ 2.0 Å.

Ligands identified through LC-MS/MS analysis were converted into three-dimensional structures, followed by hydrogen atom addition and geometry optimization using the MMFF94 force field. The minimized structures were subsequently saved in PDB format for docking simulations. Docking calculations were performed using Gnina, a deep learning–enhanced molecular docking software derived from Smina and AutoDock Vina. Gnina is publicly available via GitHub and was executed on Google Colab utilizing GPU runtime acceleration. The docking grid parameters were defined using AutoDockTools v1.5.7. Grid box dimensions were manually configured via the “--center” and “--size” parameters to ensure accurate ligand positioning within the active site. Docking results were prioritized based on binding affinity values, with lower binding free energy (BFE) indicating more favorable ligand–protein interactions. The molecular interactions of the top-ranked docking complexes were further analyzed and visualized using BIOVIA Discovery Studio to characterize hydrogen bonding, hydrophobic contacts, and other key interaction features [17].

### Molecular dynamics simulation

To evaluate the stability of the interaction between the active compound and the α-glucosidase enzyme, molecular dynamics (MD) simulations were performed using Amber20 software with GPU acceleration. The protein model was encapsulated in an explicit aqueous environment using a solvent box extending 10 Å from the solute, with the addition of Na⁺ and Cl⁻ ions to mimic physiological conditions at a salt concentration of 0.15 M. Protein parameters were assigned based on the ff19SB force field, while the ligand was parameterized using GAFF2 with atomic charges calculated through the AM1-BCC method.

The simulation began with a two-step energy minimization process: an initial phase with positional restraints of 25 kcal/mol·Å², followed by a second phase with reduced restraints of 5 kcal/mol·Å². Subsequently, the system was gradually heated to 300 K under constant volume and temperature (NVT) conditions over 50 picoseconds, then equilibrated under constant pressure and temperature (NPT) conditions to adjust pressure and density, targeting 1 g/cm³.

During the production phase, positional restraints on the complex were incrementally released every 50 picoseconds until a 500-ns unrestrained simulation was conducted at 300 K. Long-range electrostatic interactions were accurately computed using the Particle-Mesh Ewald (PME) method, and hydrogen bond lengths were constrained using the SHAKE algorithm. Temperature and pressure control were maintained using the Langevin thermostat and Berendsen barostat, respectively, to approximate realistic biological conditions [18–20].

### ADMET prediction

ADMET analysis (Absorption, Distribution, Metabolism, Excretion, and Toxicity) was performed using the pkCSM web server (http://biosig.unimelb.edu.au/pkcsm). Absorption factors include water solubility, Caco-2 permeability, intestinal absorption, P-glycoprotein substrate, P-glycoprotein I and II inhibitors, to predict compound distribution, including blood-brain barrier (BBB) permeability, central nervous system (CNS) permeability. Computational metabolism assessment involves identifying compounds as substrates for CYP2D6, CYP3A4, and their potential as inhibitors for CYP1A2, CYP2C19, CYP2C9, CYP2D6, and CYP3A4. Criteria for evaluating excretion include total clearance. To assess the toxicity profile, pkCSM is used to predict the AMES toxicity and hepatotoxicity levels as well as the effectiveness of the compound as an inhibitor of hERG I and II [21].

## Result and discussion

### Antioxidant and α-glucosidase inhibitory activity of *E. crista-galli* stem bark

Diabetes mellitus is closely related to reactive oxygen species and postprandial hyperglycaemia [22]. Excessive production of reactive oxygen species (ROS) impairs insulin secretion and sensitivity [23]. On the other hand, rapid carbohydrate digestion elevates blood glucose levels [24]. Therefore, the antioxidant and α-glucosidase inhibitory activity were evaluated in order to uncover the antidiabetic potential of *E. crista-galli* stem bark. The result showed that ethanol extract exhibited very strong antioxidant activity with an *IC*_50_ of 5.81 $\:\pm\:$ 0.49 µg/mL against DPPH free radicals and significant α-glucosidase inhibitory activity with an *IC*_50_ of 19.03 $\:\pm\:$ 0.02 µg/mL. Quercetin as a positive control, exhibited an *IC*_50_ of 7.47 ± 0.07 µg/mL in the DPPH assay and 3.93 $\:\pm\:$ 0.20 µg/mL in the α-glucosidase inhibition assay. Quercetin was used as the positive control in the α-glucosidase inhibition assay because it is a well-studied flavonoid and is commonly employed as a reference when assessing the activity of plant-derived extracts. Earlier reports also show that quercetin can inhibit α-glucosidase with an effectiveness that is comparable to and in some cases greater than acarbose, which is widely used as a standard commercial inhibitor [25].

The ethanol extract was further fractionated to yield *n-*hexane, ethyl acetate, and *n-*butanol soluble fractions, which were subsequently subjected to α-glucosidase inhibition testing. The ethyl acetate fraction showed an *IC*_50_ value of 56.61 ± 3.00 µg/mL, which falls into the moderate activity category, while the *n-*hexane fraction exhibited an *IC*_50_ value of 62.02 ± 0.07 µg/mL, slightly lower than that of ethyl acetate. Weaker activity was observed in the *n-*butanol fraction, with a very high *IC*_50_ value of 898.29 ± 173.96 µg/mL, indicating low inhibitory potential. Despite the fractionation, none of these fractions surpassed the inhibitory activity of crude ethanol extract, suggesting that the strong inhibitory effect may arise from synergistic activity among multiple phytochemicals present in the crude ethanol extract. Furthermore, statistical analysis suggests that the α-glucosidase inhibitory effect of *n-*butanol soluble fraction significantly different than those of ethanol extract, ethyl acetate soluble fraction, and *n-*hexane soluble fraction (*p*-value < 0.05). In contrast, no significant differences observed among the ethanol extract, ethyl acetate soluble fraction, and *n-*hexane soluble fraction (*p* value > 0.05).

Comparison with other parts of *E. crista-galli*, the ethanol extract of *E. crista-galli* stem bark showed much higher antioxidant activity compared to the flower extract [11, 26] and twigs extract [27]. While the antioxidant properties of different plant parts have been reported, studies investigating the α-glucosidase inhibitory effect of *E. crista-galli* extract remain limited. Therefore, the present work provides initial scientific evidence for the potency of *E. crista-galli* stem bark as natural source of antioxidant and α-glucosidase inhibitors for further relevant antidiabetic research.

### Metabolite profiling of flavonoid from *E. crista galli* stem bark

The in vitro assay revealed that the ethanol extract possesses both antioxidant and α-glucosidase inhibition activity, which can be attributed to the presence of bioactive compounds in the extract. To identify secondary metabolites in the ethanol extract, LC-MS/MS analysis was performed both in positive and negative ion mode. Compound identification was carried out using molecular formulas calculated from the mass spectra and then compared with previously reported flavonoids from *Erythrina* as outlined in our previous work [11, 28]. This analysis successfully identified several bioactive compounds, primarily flavonoids (Table 1).

**Table 1:** Flavonoids identified in *E. crista-galli* stem bark

RT	Mass	Error	Molecular formula	Compound	Previously reported in	Reference
Positive ion mode						
4.18	595.1666	0.5	C_27_H_30_O_15_	Vicenin-2 **(1)**	*E. falcata*	[29]
6.62	353.1383	1.7	C_21_H_20_O_5_	Sigmoidin H **(2)**	*E. senegalensis*	[30]
7.38	369.1323	4.1	C_21_H_20_O_6_	Abyssinin I **(3)**	*E. abyssinica*	[31]
8.55	355.1542	0.8	C_21_H_22_O_5_	Cristacarpin (Erythrabissin I)** (4)**	*E. crista-galli*	[32]
8.79	345.1362	7	C_19_H_20_O_6_	6-Methoxyhamiltone A** (5)**	*E. schliebenii*	[33]
8.92	353.141	5.9	C_21_H_20_O_5_	Erybraedin F **(6)**	*E. senegalensis*	[34]
9.32	301.1096	6.6	C_17_H_16_O_5_	Eryvarin H **(7)**	*E. abyssinica*	[35]
9.84	337.1454	4.2	C_21_H_20_O_4_	Eryvarin D **(8)**	*E. abyssinica*	[35]
10.09	355.156	4.2	C_21_H_22_O_5_	Prostratol C **(9)**	*E. abyssinica*	[36]
10.44	325.1433	2.2	C_20_H_20_O_4_	Addisofuran B **(10)**	*E. addisoniae*	[37]
10.6	337.1425	4.4	C_21_H_20_O_4_	Abyssinone A **(11)**	*E. abyssinica*	[38]
10.99	321.1135	2.5	C_20_H_16_O_4_	Erythrinin A **(12)**	*E. variegata*	[39]
11.16	409.2021	1.5	C_25_H_28_O_5_	Lonchocarpol A (Senegalensein) **(13)**	*E. crista-galli*	[40]
11.52	353.1401	3.4	C_21_H_20_O_5_	Eryvarin O **(14)**	*E. variegata*	[41]
11.75	409.2026	2.7	C_25_H_28_O_5_	Erysubin D **(15)**	*E. lysistemon*	[42]
12.94	393.2076	2.5	C_25_H_28_O_4_	2ʹ,7-Dihydroxy-3ʹ -(3-methylbut-2- enyl)−2ʹʹʹ,2ʹʹʹ-dimethylpyrano [5ʹʹ,6ʹʹ:4ʹ,5ʹ]isoflavan **(16)**	*E. mildbraedii*	[43]
Negative ion mode						
4.16	593.1539	5.6	C_27_H_30_O_15_	Isovitexin-2ʹʹ-β-ᴅ- glucopyranoside **(17)**	*E. caffra*	[44]
4.38	563.1422	3.7	C_26_H_28_O_14_	Schaftoside **(18)**	*E. abyssinica*	[45]
4.79	431.0999	4.9	C_21_H_20_O_10_	Isovitexin **(19)**	*E. crista-galli*	[46]
6.51	253.0531	11.9	C_15_H_10_O_4_	Daidzein **(20)**	*E. crista-galli*	[47]
6.62	387.145	1.5	C_21_H_24_O_7_	Orientanol A** (21)**	*E. fusca*	[48]
7.61	267.0298	1.9	C_15_H_8_O_5_	Coumasterol **(22)**	*E. crista-galli*	[47]
7.94	369.1349	3	C_21_H_22_O_6_	Abyssinoflavanone II (Abyssinin II) **(23)**	*E. abyssinica*	[31]
8.45	371.1498	0.8	C_21_H_24_O_6_	3-Hydroxy-10-(2,3-dihydroxy-3- methylbutyl)−9- methoxypterocarpan **(24)**	*E. schliebeni*	[33]
8.82	299.0577	7	C_16_H_12_O_6_	3ʹ-O-Methylorobol **(25)**	*E. eriotriocha*	[49]
8.92	339.1241	2.7	C_20_H_20_O_5_	Erystagallin C **(26)**	*E. crista-galli*	[32]
10.02	321.1143	5	C_20_H_18_O_4_	Phaseollin **(27)**	*E. crista-galli*	[50]
10.42	335.0948	8.7	C_20_H_16_O_5_	Alpinumisoflavone **(28)**	*E. indica*	[51]
10.78	369.1349	3	C_21_H_22_O_6_	Erypoegin I **(29)**	*E. fusca*	[48]
11.66	407.1862	1	C_25_H_28_O_5_	2-(γ, γ-dimethylallyl)−6a-hydroxyphaseollidin **(30)**	*E. crista-galli*	[32]
12.06	391.1906	0.8	C_25_H_28_O_4_	Erythrabyssin II **(31)**	*E. crista-galli*	[32]
12.2	423.1815	1.7	C_25_H_28_O_6_	Sigmoidin A **(32)**	*E. abyssinica*	[52]
12.4	421.1662	2.6	C_25_H_26_O_6_	Sigmoidin F **(33)**	*E. abyssinica*	[31]
12.98	407.1881	5.6	C_25_H_28_O_5_	Demethylerystagallin A **(34)**	*E. crista-galli*	[32]
13.24	405.1718	3.9	C_25_H_26_O_5_	6,8-Diprenylgenistein **(35)**	*E. crista-galli*	[50]
14.08	405.1702	0	C_25_H_26_O_5_	Bidwillon B **(36)**	*E. Orientalis*	[53]

A flavonoid was detected at a retention time of 4.18 min with an *m/z* of 595.1666 (molecular formula C_27_H_29_O_15_ [M-H]^−^, mass error 0.5). The MS/MS spectrum of the *m/z* 595.166 ion shows the characteristic fragmentation pattern of C-glycoside flavonoids. The main peaks at *m/z* 325 and 295 result from the loss of two sugar groups, yielding the apigenin aglycone and its RDA fragment. Small peaks at *m/z* 127 and 121 indicate the presence of the characteristic oxygenated aromatic ring in flavones. This molecular formula corresponds to vicenin-2 **(1)**, as previously reported in *E. falcata* [29]. Vicenin-2 **(1)** is known to reduce hyperglycaemia by inhibiting glucosidase enzymes, decrease insulin resistance via PTP1B inhibition, and alleviate complications related to diabetes mellitus by inhibiting the AR and AGE pathways [54]. At RT 6.51 min, a signal at *m/z* 253.0531 (C_15_H_9_O_4_ [M-H]⁻) corresponded to Daidzein **(20)**. This tentative identification is further supported by MS/MS spectrum of the ion at *m/z* 253.05 and the fragmentation pattern exhibited diagnostic ions at *m/z* 152 and 137, corresponding to A- and B-ring fragments, consistent with typical isoflavone fragmentation behavior [55]. These results are in good agreement with previously reported MS/MS data for Daidzein **(20)**. Daidzein has α-glucosidase inhibitor with an *IC*_50_ of 15.7 ± 0.3 µM [47].

In addition, several compounds with reported PTP1B inhibitory activity were detected, including Abyssinin I **(3)** at RT 7.38 min (C_21_H_19_O_6_ [M-H]⁻; *IC*_50_ = 18.2 ± 1.4 µM) [31]. The MS/MS spectrum of the precursor ion at *m/z* 369.1323 ([M + H]⁺) further confirms the identification of Abyssinin I as a prenylated flavanone. The fragmentation pattern shown by the characteristic product ion at *m/z* 357 indicates the cleavage of the prenyl substituent. Prostratol C **(9)** at RT 10.09 min (C_21_H_21_O_5_ [M-H]⁻; *IC*_50_ = 17.2 ± 1.6 µM) [36]. In MS/MS fragmentation, there are characteristics at *m/z* 351 and 323, which correspond to the sequential loss of hydroxyl and methoxyl groups [56]. Addisofuran B **(10)** at RT 10.44 min (C_21_H_19_O_4_ [M-H]⁻; *IC*_50_ = 15.7 ± 1.6 µM) [37]. This is supported by the MS/MS spectrum showing fragment ions at *m/z* 299, 307, 321, and 323, which reflect typical neutral losses such as H_2_O and CO, indicating a flavonoid fragmentation pattern [56]. Other identified inhibitors included Erysubin D **(15)** (RT 11.75 min; C_25_H_27_O_4_ [M-H]⁻; *IC*_50_= 9.7 ± 0.15 µg/mL) [42] and 2′,7-dihydroxy-3′-(3-methylbut-2-enyl)−2′′′,2′′′-dimethylpyrano[5′′,6′′:4′,5′]isoflavan **(16)** (RT 12.94 min; C_25_H_27_O_5_ [M-H]⁻; *IC*_50_= 5.5 ± 0.3 µM) [43].

Additional compounds with reported PTP1B inhibitory activity were Abyssinoflavanone II **(23)** (RT 7.94 min; *IC*_50_ = 17.3 ± 1.4 µM) [42], Erystagallin C **(26)** (RT 8.92 min; *IC*_50_ > 30 µg/mL) [32], dan Phaseollin **(27)** (RT 10.02 min; *IC*_50_ = 15.1 ± 1.2 µg/ml) [39, 47]. Erythrabyssin II **(31)** (RT 12.06 min; *IC*_50_> 30 µg/ml) [32, 57], Sigmoidin A **(32)** (RT 12.2 min; *IC*_50_ = 14.4 ± 0.8 µM), dan Sigmoidin F **(33)** (RT 12.4 min; *IC*_50_ = 14.2 ± 1.7 µM) [42]. Protein Tyrosine Phosphatase 1B (PTP1B) plays a critical role in the pathogenesis of type 2 diabetes mellitus (T2DM) by acting as a negative regulator of insulin receptor signaling pathways. Overactivity of PTP1B decreases phosphorylation levels of the insulin receptor, thereby weakening insulin signaling and promoting insulin resistance. Therefore, inhibition of PTP1B using specific inhibitors is considered a potential therapeutic strategy to enhance insulin sensitivity [58].

Interestingly, several flavonoids exhibited dual activity as both α-glucosidase and PTP1B inhibitors. At RT 9.32 min, a compound with *m/z* 301.1096 (C₁₇H₁₅O₅ [M-H]⁻, mass error 6.6) was identified as Eryvarin H **(7)**, reported to inhibit PTP1B and α-glucosidase with *IC*_50_ values of 8.03 ± 0.16 µM and 68.96 ± 0.74 µM, respectively [13]. Similarly, Alpinumisoflavone **(28)**, detected at RT 10.42 min (*m/z* 335.0948; C_20_H_15_O_5_ [M-H]⁻, mass error 8.7), has been shown to inhibit PTP1B (*IC*_50_ = 21.2 µM) and α-glucosidase (*IC*_50_ = 73.3 µM) [51, 59, 60]. Such dual activity suggests a synergistic effect in glycemic control, combining suppression of postprandial glucose spikes with improved insulin sensitivity, thereby strengthening their potential as promising antidiabetic candidates. At RT 4.79 min, isovitexin **(19)** was detected (*m/z* 431.0999; C_21_H_19_O_10_ [M-H]⁻) [46]. Previous studies demonstrated that isovitexin significantly reduces postprandial blood glucose levels in both normoglycemic mice and sucrose-induced diabetic rats. Notably, this effect was observed at an oral dose of 100 mg/kg, with no signs of toxicity even at 2 g/kg, supporting its safety and therapeutic potential as a natural antidiabetic agent [61].

Collectively, these findings highlight the presence of multiple bioactive compounds in *E. crista-galli* stem bark extract with complementary mechanisms of action, including α-glucosidase inhibition, PTP1B inhibition, and dual activity. This multi-target profile underscores the therapeutic potential of the extract in managing T2DM. To further validate these observations, in silico analyses including molecular docking and molecular dynamics simulations, were performed to elucidate binding interactions and stability of the identified compounds with α-glucosidase.

### Molecular docking

To explore the molecular basis of the observed α-glucosidase inhibitory activity, molecular docking was performed using the crystal structure of isomaltase from *Saccharomyces* *cerevisiae* (PDB ID: 3A4A; resolution 1.60 Å). This structure shares approximately 84% sequence similarity with α-glucosidase from *S. cerevisiae*, making it a reliable structural model [62]. Importantly, the in vitro inhibitory assay in this study also employed α-glucosidase derived from *S. cerevisiae*, ensuring biological and methodological consistency between experimental and computational approaches.

**Fig. 1:**
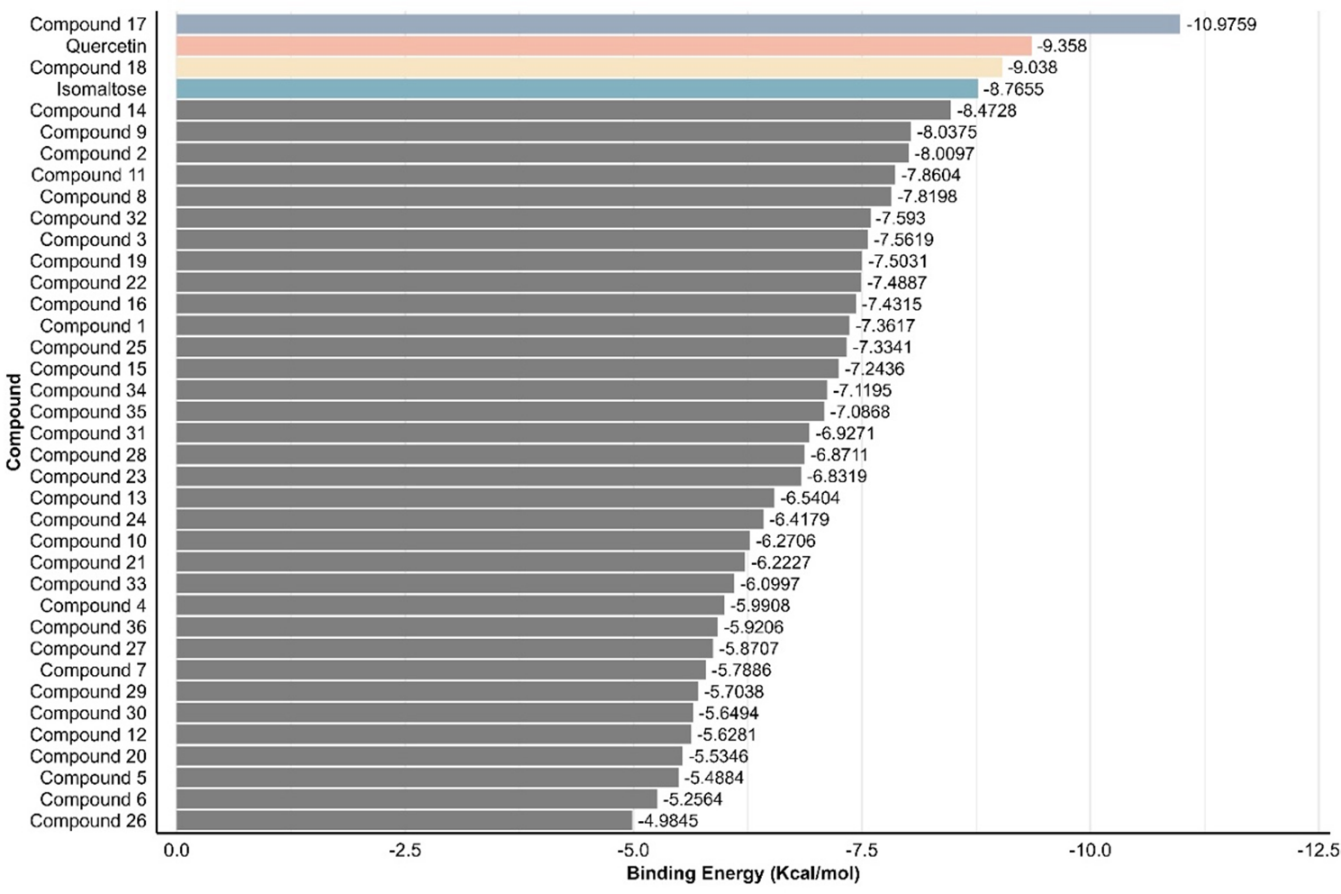
Binding affinity values of identified compounds from *E. crista-galli* stem bark against α-glucosidase (PDB ID: 3A4A) obtained from molecular docking analysis

All 36 flavonoids identified by LC–MS/MS were subjected to docking against the 3A4A active site and compared directly with the natural substrate, isomaltose (Fig. 1). Among all evaluated compounds, compound **17** (isovitexin-2’’-β-D-glucopyranoside) demonstrated the strongest predicted binding affinity (−10.9759 kcal/mol), followed by compound **18** (schaftoside) with −9.038 kcal/mol. Notably, both compounds exhibited stronger binding energies than the natural substrate isomaltose, suggesting a higher stability of the ligand–enzyme complex. Furthermore, compound **17** showed a more favorable binding affinity than the positive control quercetin, indicating its potential as a promising α-glucosidase inhibitor.

**Fig. 2:**
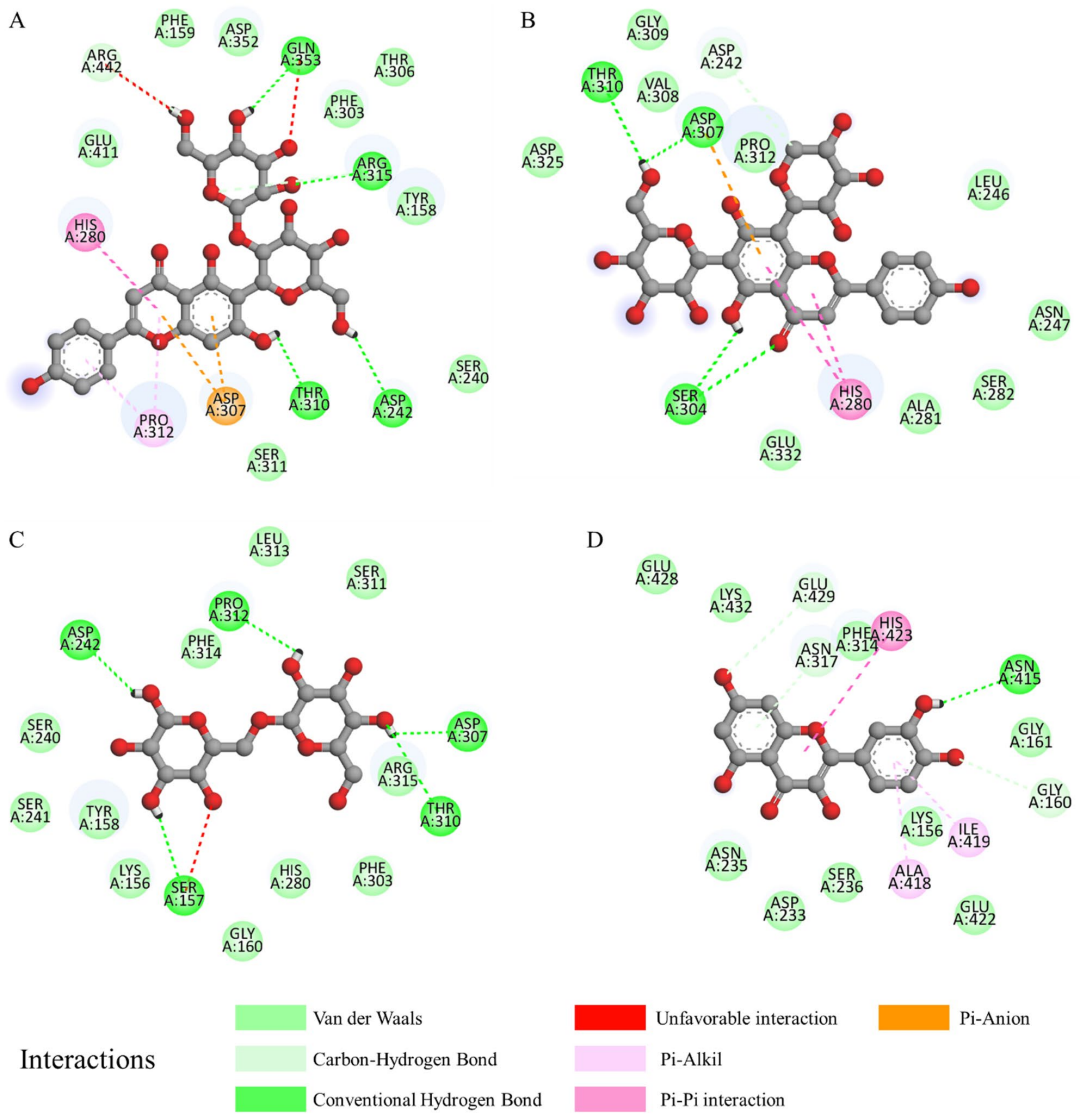
2D interaction diagrams of compound **17** (**a**), compound **18** (**b**), isomaltose (**c**), and quercetin (**d**) within the active site of α-glucosidase (PDB ID: 3A4A)

The binding interaction analysis revealed notable differences between the top-performing flavonoids and the natural substrate, isomaltose. Compound **17** exhibited multiple conventional hydrogen bonds with key active-site residues, including ASP242, THR310, ARG315, and GLN353 (Fig. 2). In addition, a $\:{\uppi\:}$-anion interaction with ASP307 and $\:{\uppi\:}$-$\:{\uppi\:}$ stacking interactions were observed, contributing to enhanced stabilization within the catalytic pocket. The combination of polar and aromatic interactions likely accounts for the superior binding affinity of this ligand.

Similarly, compound **18** formed hydrogen bonds with THR310, ASP307, and SER304, along with $\:{\uppi\:}$-$\:{\uppi\:}$ stacking interaction with HIS280. The presence of both hydrogen bonding and aromatic interactions suggests a stable binding mode within the substrate-binding region, potentially supporting competitive inhibition.

In contrast, isomaltose predominantly interacted with the enzyme through hydrogen bonding and lacked aromatic-based stabilization due to its non-aromatic structure. Moreover, the presence of an unfavorable acceptor-acceptor interaction may further reduce its binding stability. These interaction characteristics are consistent with its comparatively weaker binding energy. Quercetin demonstrated moderate stabilization through hydrogen bonding and $\:{\uppi\:}$-$\:{\uppi\:}$ interactions; however, the interaction network was less extensive than that observed for compound **17** and compound **18**. Overall, the enhanced binding affinity of selected flavonoids appears to arise from the synergistic contribution of hydrogen bonding and π-mediated interactions within the catalytic cavity. These findings provide mechanistic insight into how specific flavonoid structures may confer stronger inhibition relative to the natural substrate.

### Molecular dynamics simulation

**Fig. 3:**
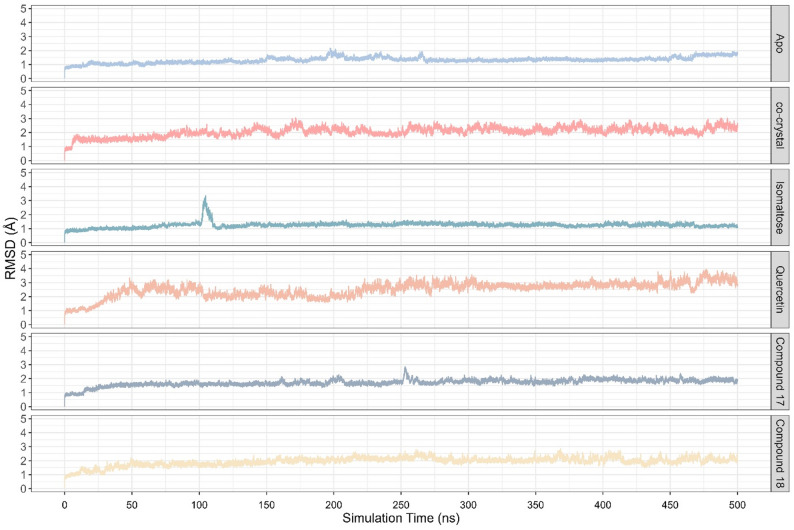
RMSD profile from 500 ns MD simulation

Molecular dynamics (MD) simulation was conducted to evaluate the binding stability of compound **17** and **18**, with comparison to that of isomaltose, quercetin, and the co-crystallized ligand through several complimentary analyses, including RMSD, hydrogen bond, and Molecular Mechanics/Generalized Born Surface Area (MMGBSA) free energy calculation. From the RMSD analysis (Fig. 3), the complex of α-glucosidase with isomaltose, which is the substrate of α-glucosidase showed the lowest RMSD value with median of 1.246 ± 0.114 Å, which is lower than that of the apo form (RMSD = 1.320 ± 0.163 Å), indicating that the enzyme-substrate complex maintained the most stable complex. Compound **17** and **18** both exhibited slightly elevated RMSD with values of 1.725 ± 0.192 Å and 1.979 ± 0.240 Å, respectively, indicating a slightly greater structural fluctuation of the α-glucosidase upon binding to these compounds. The co-crystallized ligand showed the highest RMSD value of 2.640 ± 0.445 Å, but still considered stable, as the RMSD value bellow 3 Å [63].

**Fig. 4:**
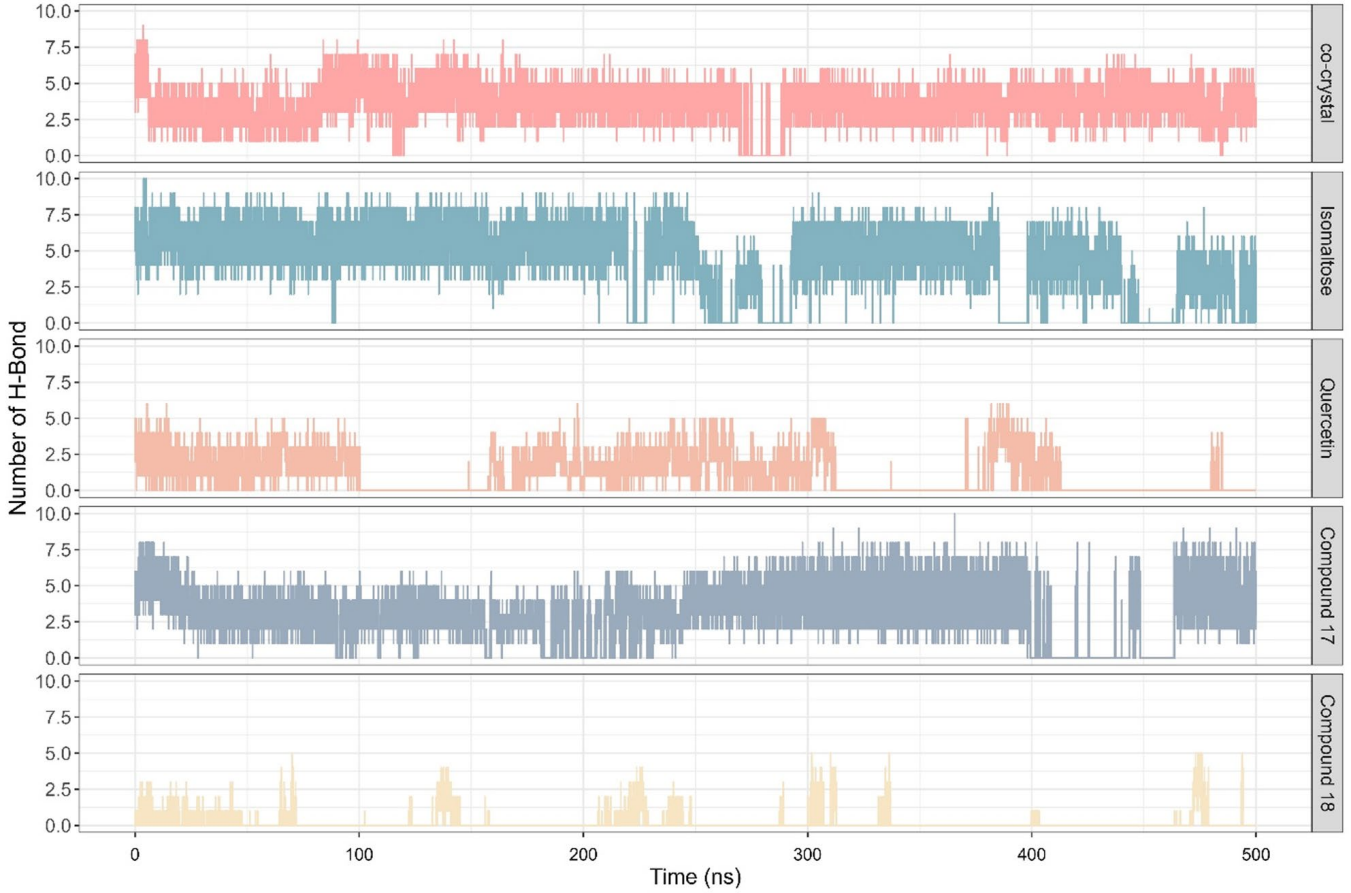
Hydrogen bond profile from 500 ns MD simulation

Hydrogen bond analysis revealed that α-glucosidase in complex with isomaltose, co-crystallized ligand, and compound **17** exhibited the highest number of hydrogen bond (HB) (Fig. 4). The complex of α-glucosidase with isomaltose formed the greatest number of hydrogen bond, reaching maximum of 10 hydrogen bonds with median of 5 hydrogen bonds. This is followed by the co-crystallized ligand complex with maximum hydrogen bond of 9 and median of 4, whereas α-glucosidase in complex with compound **17**, while also achieving maximum of 10 hydrogen bonds, showed lower median of 3.

MMGBSA free energy calculation (Table 2) positioned compound **17** as the most promising candidate with the lowest binding energy (−32.78460 kcal/mol), compared to both the co-crystallized ligand and quercetin (positive control used for in vitro assay). The low binding energy of α-glucosidase-compound **17** complex primarily driven by favourable van der Waals (−48.27415 kcal/mol) and electrostatic (−64.52990 kcal/mol) interactions, which offset the desolvation penalty (86.74850 kcal/mol). Furthermore, energy decomposition analysis revealed that compound **17** engages with multiple important amino acids of the α-glucosidase active sites, TYR-158 (−4.751 kcal/mol), HIS-280 (−4.882 kcal/mol), ARG-315 (−7.2785 kcal/mol), PRO-312 (−4.293 kcal/mol), PHE-314 (−1.9725 kcal/mol) which are part of the entrance to the active site pocket of isomaltose, as well as GLU-277 (−4.7405 kcal/mol) and ASP-352 (−0.502 kcal/mol) which are part of the catalytic residues [64].

**Table 2:** Mmgbsa free binding energy

Complex	ΔG^o^_MMGBSA_ (kcal/mol)	ΔG^o^_MMGBSA_ Energy Terms (kcal/mol)			
		VDW	EEL	EGB	ESF
Co-crystallized ligand	−5.88150	−11.15725	−63.34310	71.54725	−3.59070
Isomaltose	−25.50275	−25.36775	−76.16940	83.50240	−5.74700
Quercetin	−17.96795	−24.78850	−28.58495	40.03290	−4.02290
Compound **17**	−32.78460	−48.27415	−64.52990	86.74850	−7.74080
Compound **18**	−9.85500	−20.05885	−16.00335	28.18815	−2.57010

### ADMET prediction

A rigorous evaluation of bioavailability parameters, pharmacokinetic profile, including absorption, distribution, metabolism, and excretion (ADME), and toxicological properties of the top-performing phytochemical compounds identified through molecular dynamics simulations was conducted using the widely recognized computational tool, pkCSM (Table 3).

**Table 3:** Predicted ADMET profile of isovitexin-2’’-β-D-glucopyranoside obtained from in silico pharmacokinetic and toxicity analysis

Pharmacokinetic property	Parameters	Predicted values
Absorption	Water solubility	−2.855 log mol/L
	Caco-2 permeability	−0.59 log cm/s
	Intestinal absorption	28.656%
	P-glycoprotein substrate	Yes
	P-glycoprotein I inhibitor	No
	P-glycoprotein II inhibitor	No
Distribution	BBB permeability	−2.371 log BBB
	CNS permeability	−5.656 log PS
Metabolism	CYP2D6 substrate	No
	CYP3A4 substrate	No
	CYP1A2 inhibitor	No
	CYP2C19 inhibitor	No
	CYP2C9 inhibitor	No
	CYP2D6 inhibitor	No
	CYP3A4 inhibitor	No
Excretion	Total clearance	0.281 log ml/min/kg
Toxicity	AMES toxicity	No
	hERG I inhibitor	No
	hERG II inhibitor	Yes
	hepatotoxicity	No

The pharmacokinetic and toxicity profiles of isovitexin-2’’-β-D-glucopyranoside **(17)** were evaluated using an in silico ADMET prediction approach. The compound exhibited moderate aqueous solubility (−2.855 log mol/L) and low Caco-2 permeability (−0.59 log cm/s), with a predicted intestinal absorption of 28.656%. These findings suggest limited but plausible oral bioavailability. Moreover, the observed low Caco-2 permeability value (< 0.9) indicates limited systemic circulation penetration, aligning well with the mechanistic action of α-glucosidase inhibitors, which predominantly exert their effects within the gastrointestinal tract [65]. The compound was predicted to be a P-glycoprotein substrate, although it did not inhibit P-glycoprotein I or II, indicating possible efflux susceptibility without transporter inhibition liability.

From a distribution perspective, both BBB permeability and CNS permeability values were categorized as low, indicating that the compound is unlikely to penetrate the central nervous system. This characteristic does not represent a limitation, given that the inhibitory mechanism of α-glucosidase is localized within the gastrointestinal tract. Regarding metabolic interactions, the compound was predicted to be neither a substrate nor an inhibitor of major cytochrome P450 isoforms (CYP2D6, CYP3A4, CYP1A2, CYP2C19, and CYP2C9), suggesting a low potential for metabolic drug–drug interactions [66].

In terms of excretion, clearance serves as a crucial parameter representing the ratio between the drug concentration within the body and its rate of elimination [65]. A lower clearance value corresponds to greater drug retention in the system. The results indicate that ligand **17** exhibits a low clearance index, substantiating its prolonged persistence within the human body. Toxicological predictions showed no AMES mutagenicity or hepatotoxicity risk. However, the compound was predicted to inhibit hERG II, which may indicate a potential cardiotoxicity liability and warrants further in vitro electrophysiological validation [67].

Overall, despite moderate absorption and the predicted hERG II interaction, the compound demonstrates a generally favorable pharmacokinetic and safety profile that supports its further development as a potential intestinal α-glucosidase inhibitor.

## Conclusion

The ethanol extract of *E. crista-galli* exhibited high antioxidant activity and potent α-glucosidase inhibitory effect. The observed bioactivity may be associated with the presence of structurally diverse flavonoids identified in the in the ethanol extract of *E. crista-galli* stem bark by LC-MS/MS analysis. Furthermore, integration of molecular docking, molecular dynamics simulation, and ADMET prediction identified Isovitexin-2ʹʹ-β-ᴅ- glucopyranoside (**17**) as the predominant contributor to the α-glucosidase inhibitory activity of the ethanol extract of *E. crista-galli* stem bark with acceptable solubility, absorption, and a low predicted toxicity profile. This work provides a scientific foundation for further exploration of this plant and its constituent flavonoids in the development of nutraceutical or pharmaceutical strategies for diabetes management.

## Data Availability

All data generated or analysed during this study are included in this published article.
